# Reporter Alleles in hiPSCs: Visual Cues on Development and Disease

**DOI:** 10.3390/ijms252011009

**Published:** 2024-10-13

**Authors:** Gustavo Caldeira Cotta, Rachel Castro Teixeira dos Santos, Guilherme Mattos Jardim Costa, Samyra Maria dos Santos Nassif Lacerda

**Affiliations:** Laboratory of Cellular Biology, Department of Morphology, Institute of Biological Sciences, Federal University of Minas Gerais, 31270-901 Belo Horizonte, Brazil; gustavo-caldeira@outlook.com (G.C.C.); rachelctds@ufmg.br (R.C.T.d.S.); costagmj@gmail.com (G.M.J.C.)

**Keywords:** reporter alleles, human iPSCs, disease modeling, developmental biology

## Abstract

Reporter alleles are essential for advancing research with human induced pluripotent stem cells (hiPSCs), notably in developmental biology and disease modeling. This study investigates the state-of-the-art gene-editing techniques tailored for generating reporter alleles in hiPSCs, emphasizing their effectiveness in investigating cellular dynamics and disease mechanisms. Various methodologies, including the application of CRISPR/Cas9 technology, are discussed for accurately integrating reporter genes into the specific genomic loci. The synthesis of findings from the studies utilizing these reporter alleles reveals insights into developmental processes, genetic disorder modeling, and therapeutic screening, consolidating the existing knowledge. These hiPSC-derived models demonstrate remarkable versatility in replicating human diseases and evaluating drug efficacy, thereby accelerating translational research. Furthermore, this review addresses challenges and future directions in refining the reporter allele design and application to bolster their reliability and relevance in biomedical research. Overall, this investigation offers a comprehensive perspective on the methodologies, applications, and implications of reporter alleles in hiPSC-based studies, underscoring their essential role in advancing both fundamental scientific understanding and clinical practice.

## 1. Introduction: From Induced Pluripotent Stem Cells (iPSCs) to Reporter Alleles

Stem cells serve as the cellular foundation of the body, giving rise to specialized cells that perform specific functions [1]. They possess an extraordinary capacity for self-renewal and differentiation [1,2], making them essential to the formation of human organs and tissues. Stem cells come in two primary forms: pluripotent and multipotent. Multipotent stem cells, capable of transforming into differentiated cells from a single germ layer, are responsible for generating specific tissues, such as bone and cartilage, both of which originate from mesenchyme [1,2,3]. On the other hand, pluripotent stem cells have the remarkable ability to differentiate into cells from all three germ layers: endoderm, mesoderm, and ectoderm. This group includes embryonic stem cells (ESCs) derived from the inner cell mass of embryos and induced pluripotent stem cells (iPSCs), both demonstrating remarkable versatility [3,4].

**Figure 1:**
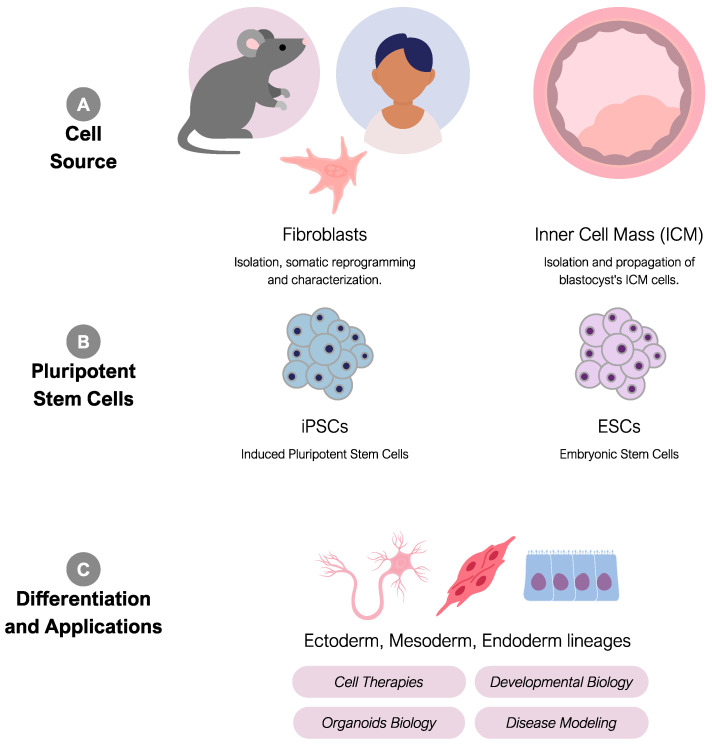
Acquisition and applications of pluripotent stem cells (PSCs). Pluripotent stem cells can be acquired through somatic reprogramming of animal or human cells, such as fibroblasts, or by collecting cells from the inner cell mass of blastocysts. Under very specific culture conditions, their pluripotent potential is maintained, enabling their use in differentiating into lineages of the three germ layers in vitro. Human PSC-derived cells have various applications, including developmental biology studies, disease modeling, organoid development, and cell therapies.

Induced pluripotent stem cells (iPSCs), first introduced by the team of Japanese researcher Shinya Yamanaka in 2006 using mouse models [5] and in 2007 using human cells [6], represent a revolutionary advancement in stem cell research. iPSCs are derived from adult specialized cells, such as dermal fibroblasts or blood cells [6,7], which are reprogrammed into a state mimicking embryonic pluripotency, completely bypassing the need for embryos (Figure 1). This reprogramming process involves the introduction of specific genes into adult cells, enabling them to regain the ability to differentiate into various cell types. The genes in question facilitate the transient expression of four key transcription factors—OCT4, SOX2, KLF4, and c-MYC, collectively known as OSKM, which orchestrate the pluripotency of iPSCs [6,8]. OCT4 and SOX2 are key transcription factors that uphold pluripotency and foster the development of diverse cell lineages. KLF4 regulates gene expression and aids in maintaining pluripotency, while c-MYC contributes to the reprogramming process and sustains pluripotency. These four transcription factors are sufficient to transform human fibroblasts into iPSCs [9]. Other transcription factors, like NANOG and LIN28, have also been recognized as significant regulators of pluripotency in iPSCs [10].

The discovery of human iPSCs (hiPSCs) marked a significant advancement by circumventing ethical concerns tied to embryonic stem cell acquisition [11]. This is chiefly because iPSCs and ESCs share numerous phenotypic markers, such as morphology and surface protein expression [12,13,14], as well as epigenetic landscapes [15]. Consequently, iPSCs exhibit crucial characteristics like self-renewal and the capacity to differentiate into diverse cell lineages, including germ cells, rendering them indispensable to various fields like regenerative medicine, disease modeling, drug discovery, and studies in developmental biology [16,17]. In the realm of regenerative medicine, iPSCs technology offers the potential to generate patient-specific cells and tissues for transplantation, mitigating the risk of immune rejection [18]. In this same way, hiPSCs serve as a versatile tool for disease modeling by helping investigate a wide spectrum of conditions, including neurological and neurodegenerative diseases [19], such as Alzheimer [20], as well as heart, kidney, and lung diseases [21,22,23]. This enables a deeper understanding of the disease mechanisms and opens avenues for potential therapeutic interventions [24]. As for disease modeling, iPSCs play a determining role in toxicity testing and drug screening and discovery, mainly by facilitating the creation of disease-specific cell lines and allowing the assessment of new drug efficacy [25]. Furthermore, iPSCs significantly contribute to developmental biology by helping elucidate the processes occurring in early embryonic states, enabling scientists to replicate these mechanisms in vitro and advance research in the aforementioned areas [26].

Developmental biology encompasses a range of intricate processes, including tissue patterning, tissue growth, cell differentiation, and morphogenesis. iPSCs, due to their inherent properties, serve as an ideal model system for investigating these processes in vitro [27]. Stem cells play a crucial role in both normal development and maintenance of adult tissues, generating diverse cell types within embryos or tissues [28]. In the context of developmental biology, iPSCs offer a valuable tool for unraveling the molecular mechanisms and signaling pathways that govern cell differentiation; scientists can direct iPSCs to differentiate into various cell types, providing insights into the complex interplay among genes, environment, and extracellular matrix components [29]. Employing iPSCs in conjunction with developmental principles enables researchers to explore the interplay between genetic and environmental factors in determining cell fate, contributing to a deeper understanding of human and animal development.

The genetic behavior of iPSCs exhibits variability under different experimental conditions, influencing the metabolic triggers and impacting nucleus dynamics, specifically in gene expression [30]. Gene expression patterns, fundamental to developmental biology, govern the processes that regulate growth, differentiation, and morphogenesis in organisms. Studying these patterns sheds light on how genes are activated or repressed during development, guiding cells to acquire distinct identities and functions [31,32]. By simultaneously examining the expression of numerous genes, researchers can identify patterns underlying cellular differentiation and tissue development. These patterns are shaped by specific combinations of transcription factors and the varied accumulation of gene products in cell subsets during development [33,34,35,36]. Understanding the gene expression patterns is essential for deciphering the intricate regulatory networks controlling the formation of functional cell types and the three-dimensional structure of tissues and organs [37,38].

Studying gene expression patterns is particularly pertinent to the subject of iPSCs. It allows researchers to track the activation and repression of specific genes during the reprogramming of adult cells into pluripotent stem cells and their subsequent differentiation into various cell lineages [39]. This understanding is essential for optimizing reprogramming and differentiation protocols, as well as uncovering molecular similarities and distinctions between iPSCs and their embryonic counterparts. In this regard, reporter alleles are invaluable tools, allowing the visualization of these dynamic processes and enhancing our comprehension of how different stimuli influence cell fate and behavior in vitro. Furthermore, reporter-gene-based molecular imaging offers a powerful approach to studying the physiology and fate of iPSC-derived cells in vivo, providing deeper insights into their functional roles in real time. Also referred to as reporter genes, these genes encode detectable products not endogenously expressed in the organism or cell type [40]. Serganova and Blasberg [41] describe the following two main types of reporter genes: constitutively expressed reporters, which provide continuous signals for long-term cell tracking, and inducible reporters, which activate in response to specific signals, allowing a detailed study of particular tissues or pathways. This work also discusses the evolution of these systems, including the use of fluorescent-, bioluminescent-, and radionuclide-based reporters, while acknowledging ongoing challenges in the field. Incorporated into DNA constructs, reporter allele products serve as visual indicators for the precise localization of specific proteins, cell types, or biological circuits [42,43].

**Figure 2:**
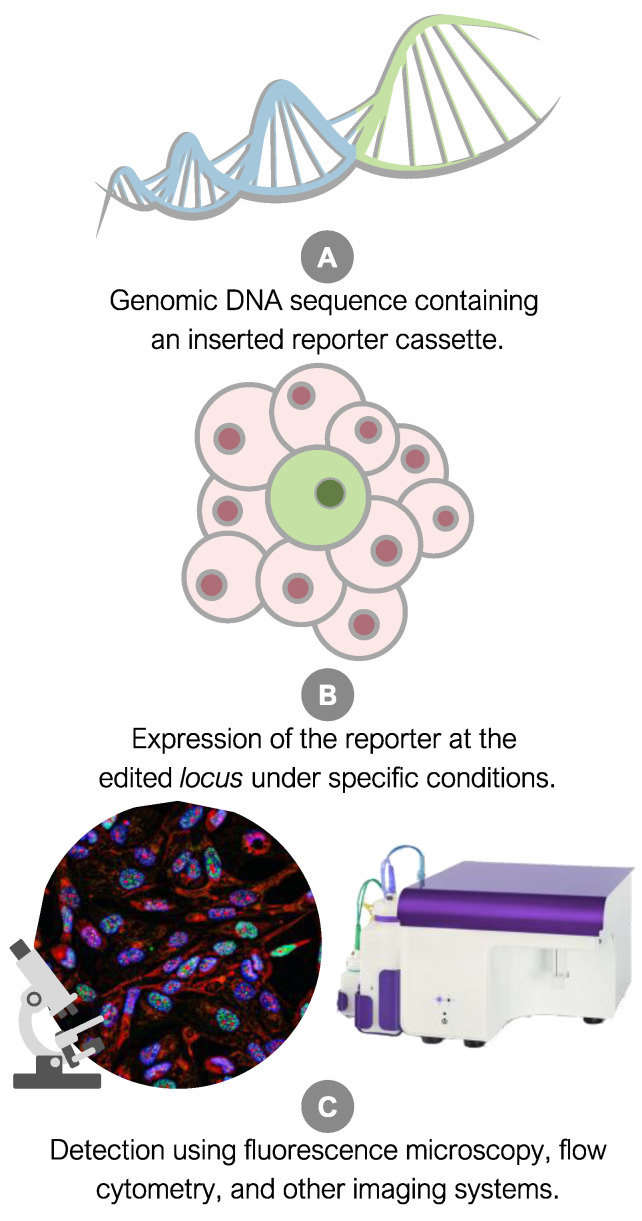
Summary of the application of fluorescent reporter alleles for the detection of specific genes (markers) using cellular imaging techniques.

Widely used reporter genes, such as green fluorescent protein (GFP) and luciferase, offer visual and quantifiable cues [44], providing insights into the spatial and temporal patterns of gene activity. Reporter alleles play an essential role in monitoring pluripotency and differentiation, allowing the assessment of pluripotency and tracking iPSC differentiation into specific cell lineages by observing key markers like OSKM [45]. Integration into specific genomic loci enables the tracking of genomic stability and assessment of iPSC genome integrity [46], contributing to quality control and characterization by evaluating specificity, purity, and differentiation potential. Furthermore, reporter alleles within iPSCs function as valuable instruments for investigating regulatory mechanisms and developmental processes involved in the differentiation of tissues and organs. The incorporation of reporter genes into the target organism or cell type involves cellular and molecular biology techniques, such as transfection with cationic lipids, electroporation, or viral transduction [47]. The subsequent surveillance of fluorescent reporter gene expression employs a diverse array of methodologies, including fluorescence/confocal microscopy, luminescence assays, and flow cytometry (Figure 2). Additionally, diverse induction protocols can be applied to monitor lineage specification from iPSCs, aiming to achieve multiple objectives and tracking numerous markers relevant to the differentiation process.

Against this background, this review will highlight key methodologies for creating hiPSC lines carrying reporter alleles and explore their primary applications in developmental biology and disease development comprehension, presenting studies and potential advancements in these research areas.

## 2. Strategies for Introducing Reporter Alleles in hiPSCs

**Figure 3:**
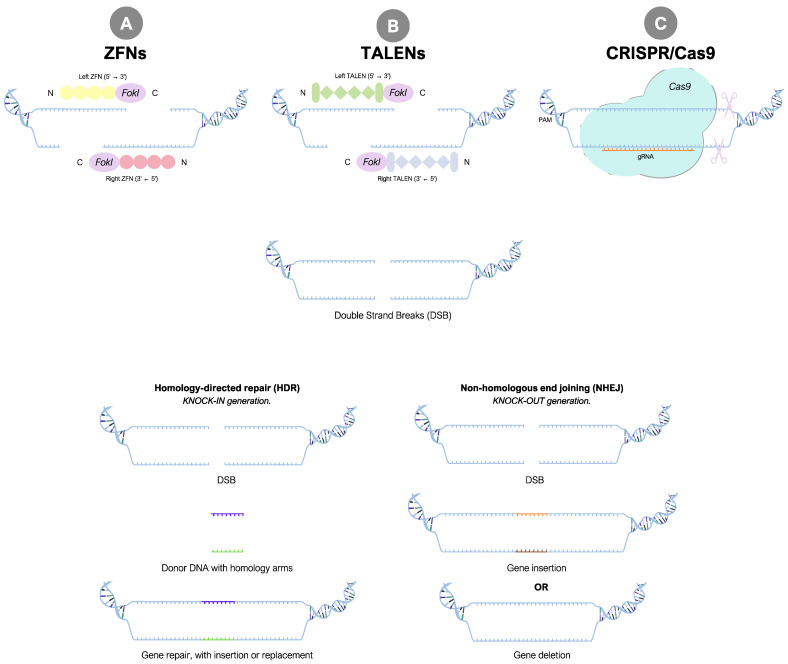
Overview of the three major gene-editing platforms. ZFNs, TALENs, and CRISPR/Cas9 rely on the HDR pathway.

Gene editing has revolutionized the ability to precisely modify the genetic information within cells [48]. It involves the targeted addition, deletion, or modification of DNA sequences, enabling researchers to study gene functions, model diseases, and potentially develop therapeutic interventions [49]. One particularly impactful application of gene editing is the generation of cells carrying reporter genes [50], the core of our interests. This process involves integrating reporter genes—which are determined by the researcher’s direction in accordance with their objectives—into the cellular genome, providing a powerful tool for studying promoter-driven transcriptional activity, cellular responses, and various biological phenomena [51]. Several gene-editing methodologies can be applied to generate reporter cell lines, with a predominant reliance on homologous recombination (HR), a type of homology-directed repair (HDR). The double-strand DNA break (DSB), induced by an exogenous nuclease, immediately triggers the cell to activate a DNA repair pathway, such as non-homologous end joining (NHEJ) or HDR (Figure 3).

The selection between NHEJ and HDR is largely influenced by DNA end resection, a nucleolytic process in which DSB ends are converted into 3′ single-stranded DNA (ssDNA) overhangs. Resected DNA ends can undergo processing by fill-in or endonucleolytic cleavage, resulting in blunt ends compatible with NHEJ. These post-resection steps may introduce errors during NHEJ, such as deletions or insertions (INDELs) of ssDNA overhang fragments, which facilitate the generation of specific knock-out cell lines. Unlike NHEJ, which simply rejoins the cleaved DNA ends, the HDR pathway requires the presence of an identical or highly similar template—a fully intact sequence—to accurately repair the damaged DNA [52]. The possibility to introduce an exogenous sequence as a template (donor DNA) for use in HDR represents the key element of gene editing via the HR pathway, which is extensively explored in gene targeting. Therefore, HR plays a central role in numerous gene-editing strategies aimed at creating cells with reporter genes, utilizing the cell’s intrinsic DNA repair mechanisms to achieve precise insertion or substitution of DNA sequences at specific genomic locations [47,53]. Researchers can introduce a reporter gene by delivering a DNA template with sequences homologous to the regions flanking the DNA cleavage site, enabling the HR pathway to integrate the gene of interest into a designated genomic site [51,52,54]. This approach ensures stable reporter gene expression driven by the native regulatory elements at the intended genomic locus.

HR also enables the insertion of a reporter gene cassette flanked by positive and negative selection markers. Upon introducing this cassette into cells, researchers typically apply antibiotic selection pressure, thereby ensuring the survival of cells that have successfully incorporated the cassette [55]. This efficient selection process aids in identifying cells carrying the desired reporter gene construct. Some studies opt to forgo antibiotic enrichment selection, instead employing alternative methods such as fluorescence-based or immunomagnetic selection [56,57]. Moreover, HR can be employed to create cell lines with reporter gene expression responsive to external stimuli. The precision of HR in the targeted insertion or replacement of DNA sequences minimizes unintended mutations and ensures controlled reporter gene expression. It results in stable integration into the genome, providing reliable, long-term expression suitable for the study of chronic processes [58]. The versatility of HR allows for its adaptation to various reporter gene integration strategies and selection methods, offering flexibility for diverse experimental designs.

### 2.1. Major Features of Gene-Editing Techniques

Zinc-Finger Nucleases (ZFNs), Transcription Activator-Like Endonucleases (TALENs), and Clustered Regularly Interspaced Short Palindromic Repeats (CRISPR) represent three successive generations of nuclease-based genome-editing technologies that are applied to engineer cells with reporter alleles (Figure 3). Notably, all these methods are effective gene-editing tools for introducing reporter genes into iPSCs. ZFNs and TALENs create DSB at a specific site in the genome by using a DNA-binding domain fused to a nuclease domain. In contrast, CRISPR/Cas9 uses a guide RNA to direct a Cas9 nuclease cut on the target site [59].

Over the past 20 years, ZFNs have become crucial for genome editing by targeting specific sequences and inducing DNA breaks, which allow for precise modifications during the repair process [60]. They are composed of two primary functional domains: a customizable array of zinc finger proteins (ZFPs) that confer sequence specificity, and a non-specific cleavage domain derived from the FokI restriction enzyme (Figure 3A). Each zinc finger typically recognizes three nucleotides, allowing for the design of ZFNs that can target specific genomic sequences with high precision. Upon binding to their target DNA, ZFNs dimerize, which activates the FokI domain to cleave the DNA at the desired locus [61]. This technology has been applied across various model systems, including human cells and other organisms, facilitating accurate genomic alterations. ZFNs have revolutionized genetic engineering by offering high specificity and minimal toxicity. They are one of the earliest chimeric restriction endonucleases used for targeted genome engineering [62]. They continue to be developed and diversified, making them adaptable for various research purposes and potential therapeutic applications.

TALENs, the second generation, surpass ZFNs in terms of efficiency and specificity, requiring less intricate design and validation [63]. They demonstrate applicability in diverse scenarios, such as gene targeting, functional genomics, and disease modeling [64,65,66]. TALENs are chimeric proteins that contain two functional domains: a DNA-recognition transcription activator-like effector (TAL effector) and a nuclease domain [67] (Figure 3B). They enable targeted genome cleavage, followed by gene modification during subsequent repair. TALENs were the first easy-to-use genome-editing technology and have been used in multiple species, bringing targeted genome editing to the forefront of research [68].

CRISPR/Cas9, the third-generation nuclease genome-editing tool, is widely recognized for its versatility. The system consists of two main components: the Cas9 enzyme, which acts as “molecular scissors” to cut DNA at a location specified by a guide RNA, and the guide RNA (gRNA), which binds to Cas9 and specifies the target location for DNA cleavage (Figure 3C). This technology, adapted from a naturally occurring bacterial defense system, has garnered excitement due to its cost-effectiveness, accuracy, and efficiency compared to other genome-editing methods. The high efficiency of genome editing with the CRISPR/Cas9 system makes it possible to edit multiple targets in parallel, offering an advantageous tool for a wide range of applications in human research and gene therapy [68]. However, a potential downside of the use of CRISPR/Cas9 relates to the off-target effects, a concern less pronounced with ZFNs and TALENs, which exhibit higher specificity [69]. This issue is mainly caused by the wild-type Cas9 protein properties: Cas9 has 1368 amino acids and recognizes a relatively common NGG protospacer adjacent motif (PAM), functions optimally with 20 nt spacers, and supports relatively high levels of off-target editing [70,71]. To address these limitations, researchers have developed various Cas9 variants, each with unique characteristics. Engineered variants of Cas9, such as eSpCas9, HypaCas9, and high-fidelity Cas9, have been developed to enhance specificity and reduce the off-target effects. These modifications address the challenges associated with the standard CRISPR/Cas9 system by improving fidelity without compromising cleavage activity [70,72]. For example, dead Cas9 (dCas9), a completely inactive form, has been engineered for greater precision in the context of gene targeting, silencing, and activation, while overexpressing these variants or using lower doses of Cas9 can further improve specificity [73]. Additionally, Deep SpCas9 variants and tagmentation-based tag integration site sequencing (TTISS) methods are used to assess specificity and activity, aiding in the selection of appropriate Cas9 variants for specific sequences. Other Cas9 proteins, like SaCas9, have also been engineered to offer high fidelity and a broad PAM range, minimizing the off-target effects while maintaining efficient cleavage activity [71,74]. Notably, following the gene editing of a cell population, isolation of clones is required to separate non-edited and different types of edited cells. Consequently, single-cell cloning of iPSCs can effectively address the potential low efficiency in the genome-editing procedure and also assist in identifying clones that have the intended on-target edits without any off-target mutations.

Prime editing is a revolutionary genome-editing technology that enables precise modifications without double-strand breaks or donor DNA, thereby overcoming significant limitations of the traditional CRISPR methods [75]. This platform utilizes a catalytically impaired Cas9 protein fused to reverse transcriptase (prime editor complex—PE2), guided by prime editing guide RNA (pegRNA), which facilitates the direct insertion of genetic information without the need for a donor DNA [76]. Its “search-and-replace” mechanism allows for a variety of genetic changes, making it ideal for creating reporter cell lines to monitor gene expression [77]. Recent studies highlight prime editing effectiveness in generating human reporter cell lines, particularly in challenging primary cells and specialized lines [78]. Optimized systems, like PE4 and PE5, improve editing precision and reduce byproducts [79]. Additionally, integrating reporter systems into prime editing protocols enables the real-time monitoring of editing efficiency, essential for successful reporter line generation [80].

### 2.2. Gene-Editing Strategies in iPSCs

Since Yamanaka and colleagues first described iPSCs in 2006, various strategies have been developed for gene editing in these cells. ZFNs were used for targeted genetic editing, allowing researchers to introduce specific mutations, repair disease-associated mutations, and generate isogenic cell lines with reporter genes. For example, Pei and colleagues [81] demonstrated the effectiveness of ZFNs in achieving precise gene targeting, which enabled the creation of GFP and luciferase reporter lines in hiPSCs. Specifically designed and refined ZFN pairs were employed to target the C-terminal regions of the *GFAP* gene, an intermediate filament present in neural cells, and the *MAP2* gene, a microtubule-associated protein. In parallel, a donor vector was devised, incorporating a reporter cassette featuring a P2A peptide, a luciferase (*Nanoluc*) gene fused with a HaloTag, and a neomycin resistance gene. The positioning of this cassette was meticulously aligned with the C-terminal regions of the *GFAP* or *MAP2* genes to create lineage-specific reporters in iPSC.

On the other side, TALENs have emerged as crucial players in the development of reporter-gene-iPSC lines. The utilization of TALEN-mediated gene targeting has notably heightened the expression of transgenes and facilitated the one-step creation of dual reporter hiPSC and neural stem cell (NSC) lines, underscoring the high efficiency of TALEN-mediated HDR in generating targeted mini-gene transfer or reporter knock-in cell lines [82]. This technology stands out for its effectiveness in achieving the efficient and precise integration of reporter genes at specific loci, including the *AAVS1* and *Citrate Lyase Beta-Like (CLYBL)* safe-harbor locus. Kuhn and colleagues (2017), in their study, describe the use of TALENs to integrate a codon-optimized *CSF2RA* transgene into the *AAVS1* locus in hereditary pulmonary alveolar proteinosis (herPAP) patient-derived iPSCs. Mutations in the *CSF2RA* gene are known to impair surfactant clearance, potentially leading to the accumulation of surfactants in the alveoli, a hallmark of herPAP. The efficacy of TALEN-mediated gene editing in achieving precise knock-in modifications without compromising the differentiation potential of iPSCs was demonstrated, thereby advancing potential gene therapy applications [83]. A key advantage of TALENs and CRISPR/Cas9 is their ability to perform multiple gene edits in a single experiment, which is particularly beneficial for gene therapy applications requiring the correction of multiple mutations or the introduction of therapeutic transgenes [84]. Additionally, TALENs have played a role in generating integration-free iPSCs with reporter genes, expanding the utility of hiPSCs for disease modeling, drug testing, and the exploration of cellular differentiation [83].

The specificity and efficiency demonstrated by TALENs in mediating gene targeting render them invaluable tools for the precise and reliable creation of reporter gene iPSC lines, thereby contributing significantly to advancements in emulating various health conditions and enhancing our understanding of human and animal development [82]. However, despite their substantial contributions, the use of TALENs for generating reporter gene iPSC lines presents certain potential limitations and challenges. Firstly, TALENs, like many gene-editing techniques, carry the risk of off-target effects, resulting in unintended modifications at genomic locations similar to the target site [85,86]. This introduces inaccuracies and the possibility of unintended consequences. Efficient delivery of TALEN systems to target cells presents another challenge, with varying existing methods and certain cell types or tissues proving more resistant to successful delivery. The large size of TALENs further complicates their packaging into viral vectors for delivery, affecting the overall efficiency [87]. Limitations in the targeting range of TALENs also exist, with some genomic regions proving more challenging to access or modify compared to other gene-editing tools [88]. Achieving efficient integration of donor DNA into the target site is also a challenge, relying on the simultaneous delivery of both TALEN proteins and donor DNA into cells [89,90]. Despite these challenges, TALENs remain a valuable tool for generating reporter gene iPSC lines, and ongoing advancements in TALEN-mediated gene editing continue to enhance the efficiency and precision of this technique.

The revolutionary CRISPR/Cas9 gene-editing tool has transformed the landscape of genetic manipulation. In the realm of reporter genes, researchers utilize guide RNAs (gRNAs) to direct the Cas9 endonuclease to specific genomic locations, and it triggers DNA repair mechanisms, creating an opportunity for the integration of reporter gene sequences [91]. However, this technology also presents off-target cuts, similar to the other technologies discussed. In the context of the wild-type Cas9 approach, ZFNs and TALENs offer higher specificity, although they require more complex design and optimizations [84].

The focused nature of this approach enables controlled placement of the reporter, optimizing both its expression and function [92]. The amalgamation of CRISPR/Cas9 technology with iPSCs further amplifies their potential, facilitating the introduction of valuable reporter genes. This synergy, exemplified by CRISPR/Cas9 iPSC reporter lines, is rapidly advancing fields, from genetic disorders to regenerative medicine and cell therapies [23,24,93]. CRISPR/Cas9 technology offers significant advantages in the development of iPSC reporter lines over conventional methods. Its targeted integration minimizes off-target effects, ensuring the precise expression of the reporter genes at the desired genomic locations. The inherent versatility of the CRISPR/Cas9 platform allows for the incorporation of various reporter genes and combinations, enabling researchers to customize iPSC lines for specific research questions. Additionally, the inclusion of selection markers alongside the reporter gene streamlines the identification and isolation of successfully edited iPSCs [94].

The practical application of CRISPR/Cas9 in generating reporter iPSC lines is exemplified by various studies with human and animal approaches. For instance, researchers have utilized CRISPR/Cas9 to create a tyrosine hydroxylase (TH) reporter hiPSC line for live imaging and isolation of dopaminergic neurons for the study of Parkinson disease. The TH-GFP iPSC line was generated by introducing a *GFP* reporter gene into the *TH* locus using CRISPR/Cas9-mediated HDR. After the induction of differentiation, researchers confirmed that GFP expression faithfully mimicked endogenous TH expression in iPSC-derived dopaminergic neurons. Calcium-imaging techniques were employed to determine the intrinsic functional differences between TH-GFP-positive and TH-GFP-negative cells. The brightness of GFP allowed direct visualization of TH-expressing cells in heterogeneous cultures and enabled the researchers to isolate live GFP-positive cells through fluorescence-activated cell sorting (FACS) for further applications [95]. An excellent example of the indirect application of reporter alleles and CRISPR-Cas9 editing is the study published by Patria et al. (2020). Using a hiPSC line with the tdTomato fluorescent protein fused to the *SOX9* gene, the researchers induced heterozygous mutations at the *TRPV4* locus. The *TRPV4* gene is responsible for upregulating the expression of *SOX9* during cartilage development. Therefore, in this study, mutant iPSC lines became a valuable tool for creating an in vitro model of human chondrodysplasia [96]. The CRISPR/Cas9 system has also been employed to generate *ATP1a1* reporter iPSC lines, enhancing genome-editing efficiency and enabling the study of cellular osmolarity maintenance. Liu and colleagues’ approach involved the use of a one-step co-targeting strategy with a longer dsDNA repair template, which was found to shorten the period of the selection process and increase both HDR and INDEL rates. The Na^+^/K^+^ channel, encoded by the *ATP1A1* gene, is critical for maintaining cell osmolarity, and the disruption of its ion-exchange ability can result in various cellular dysfunctions. The study demonstrated that by co-targeting *ATP1A1* with a second locus of interest, INDEL and HDR selection efficiency was improved in two different iPSC lines [97].

Despite its transformative potential, using CRISPR/Cas9 to create iPSC reporter lines poses challenges. Concerns about off-target effects persist, necessitating thorough validation post editing. The efficiency of the technology depends on optimizing the guide RNA (gRNA) design and delivery methods [91,92]. As CRISPR/Cas9 technology continues to evolve, the potential of iPSC reporter lines will expand further. The integration of artificial intelligence (AI) and machine learning algorithms is likely to optimize gRNA design and streamline the editing processes [98].

The use of prime editing technology has emerged as a transformative method that can be further applied to generate iPSCs with reporter alleles, enabling precise genomic modifications without double-strand breaks, unlike traditional CRISPR/Cas9 techniques. This method enhances editing precision and reduces the frequency of undesired insertions and deletions, making it suitable for pluripotent stem cells [99]; however, the effectiveness remains lower. Recent studies have highlighted the effectiveness of prime editing in creating specific reporter alleles in hPSCs. Wu et al. [17] showed a robust all-in-one prime editing system in human ESC, in which *tdTomato* gene expression reported the efficient creation of both monoallelic and biallelic disease-relevant mutations. The simultaneous introduction of pegRNA and nicking sgRNA further facilitated precise genome editing, enabling the generation of isogenic hiPSC lines. Additionally, Habib et al. [100] constructed a drug-inducible, PE2-expressing hESC line and addressed prime editing for correcting a disease-related mutation in iPSCs derived from a patient with α 1-antitrypsin (A1AT) deficiency. Chen et al. [79] identified cellular factors that enhance editing efficiency by manipulating DNA mismatch repair pathways, suggesting that optimizing these mechanisms could improve reporter allele generation.

### 2.3. Strategies to Favor HDR-Based Edits and Gene Targeting

Despite the great potential of gene targeting in iPSC technology, its use is still limited due to the low frequency of HDR in somatic cells. This low frequency can be attributed to the dominance of NHEJ-mediated DNA repair, as this mechanism takes place in all cell-cycle phases. To optimize HDR efficiency and inhibit NHEJ in iPSCs, some strategies have been proposed. One of the primary approaches involves the use of small molecules that can modulate the cellular repair pathways. Liu et al. [101] discuss various methodologies to improve HDR, highlighting the role of inhibitors that specifically target NHEJ components. For instance, the use of small molecule inhibitors, such as M3814, which target the DNA-PKcs protein involved in NHEJ, has been shown to significantly increase HDR rates in iPSCs by reducing the competition from NHEJ [102]. Additionally, the inclusion of histone deacetylase inhibitors, such as trichostatin A (TSA), can further enhance HDR by promoting a more favorable chromatin environment for repair [101]. Studies indicate that p53, a well-known tumor suppressor, negatively regulates HDR by inducing cell-cycle arrest and apoptosis in response to DNA damage [103]. By temporarily inhibiting p53, researchers have observed an increase in HDR efficiency in iPSCs, allowing for more successful gene-editing outcomes [103]. This strategy must be carefully controlled to avoid long-term genomic instability, but it presents a viable method for enhancing HDR during critical editing phases.

Moreover, the availability of the donor template and the precise timing of its delivery in relation to Cas9 activity are critical for optimizing HDR. Fu et al. [102] emphasize that synchronizing the introduction of donor templates with the S/G2 phases of the cell cycle, when HDR is most active, can significantly improve editing outcomes. This synchronization can be achieved through various methods, including the use of cell cycle inhibitors that can temporarily halt cells in the desired phase, thereby increasing the likelihood of HDR over NHEJ. The design of donor templates also plays a pivotal role in HDR efficiency. Utilizing longer homology arms in donor templates has been shown to enhance the likelihood of successful integration during HDR [101]. Additionally, the incorporation of specific molecules that can favor the recruitment of repair proteins to the site of the double-strand break can further improve HDR outcomes [52].

Improving the delivery of CRISPR/Cas9 components is also relevant for maximizing genome-editing efficiency. Viral vectors, particularly adeno-associated viruses (AAVs), have been extensively studied for their ability to deliver donor templates efficiently. Martin et al. demonstrated that AAV6-mediated delivery of donor templates in conjunction with CRISPR/Cas9 ribonucleoprotein (RNP) complexes led to high rates of homologous recombination in human pluripotent stem cells [104]. This method capitalizes on the natural ability of AAVs to transduce a wide range of cell types, including hiPSCs, thus facilitating the introduction of donor DNA necessary for HR. However, the reliance on viral vectors raises safety concerns, such as potential insertional mutagenesis and immune responses [105]. Liu et al. [106] highlight that the negative charge of CRISPR components can hinder their cellular uptake due to electrostatic repulsion from the negatively charged cell membrane. To overcome this barrier, various non-viral delivery systems have been developed, including cationic liposomes and nanoparticles that can encapsulate CRISPR components and facilitate their entry into cells [107]. Recent advances in lipid nanoparticles (LNPs) have shown promise in enhancing the delivery of CRISPR/Cas9 systems. These LNPs can be engineered to improve endosomal escape, thereby ensuring that the CRISPR components reach the cytosol, where they can exert their editing effects [108]. Furthermore, the use of extracellular vesicles (EVs) as delivery vehicles has gained attention due to their biocompatibility and ability to target specific cell types [109]. Although still poorly explored in hiPSCs, these novel gene delivery approaches hold significant potential for optimizing the generation of reporter lines and enhancing their application.

Recent studies have shown that delivering CRISPR/Cas9 via electroporation using RNP complexes is highly efficient in iPSCs. Electroporation, a non-viral method, improves the uptake of CRISPR components, leading to better gene-editing outcomes compared to plasmid transfection or viral vectors. RNP complexes, being transient, reduce off-target effects and increase specificity. Ruan et al. [110] found that electroporation of Cas9 RNPs resulted in significantly higher INDEL rates—27.13% in wild-type and 88.78% in patient-derived hiPSCs—while Xu et al. [111] reported efficient, low-toxicity editing using this method. Moreover, the efficiency of electroporation in delivering CRISPR components has been corroborated by studies focusing on various cell types. For instance, Smirnikhina et al. noted that the HDR efficiency in iPSCs was lower compared to other cell types when using plasmid delivery, but RNPs showed improved outcomes in terms of gene-editing precision [112]. This suggests that while electroporation is effective, the choice of the delivery method and the form of the CRISPR components—RNPs versus plasmids—can significantly influence editing efficiency. Despite the advantages, challenges remain in optimizing electroporation parameters to maximize gene-editing efficiency. For example, while electroporation has been shown to be more efficient than lentiviral transduction in other contexts [113], the specific conditions required for iPSCs can vary. Additionally, the transient expression of RNPs necessitates careful optimization of electroporation settings to ensure that a sufficient amount of the editing machinery is delivered without compromising cell viability [114].

### 2.4. Experimental Workflow to Generate iPSC Reporter Lines

**Figure 4:**
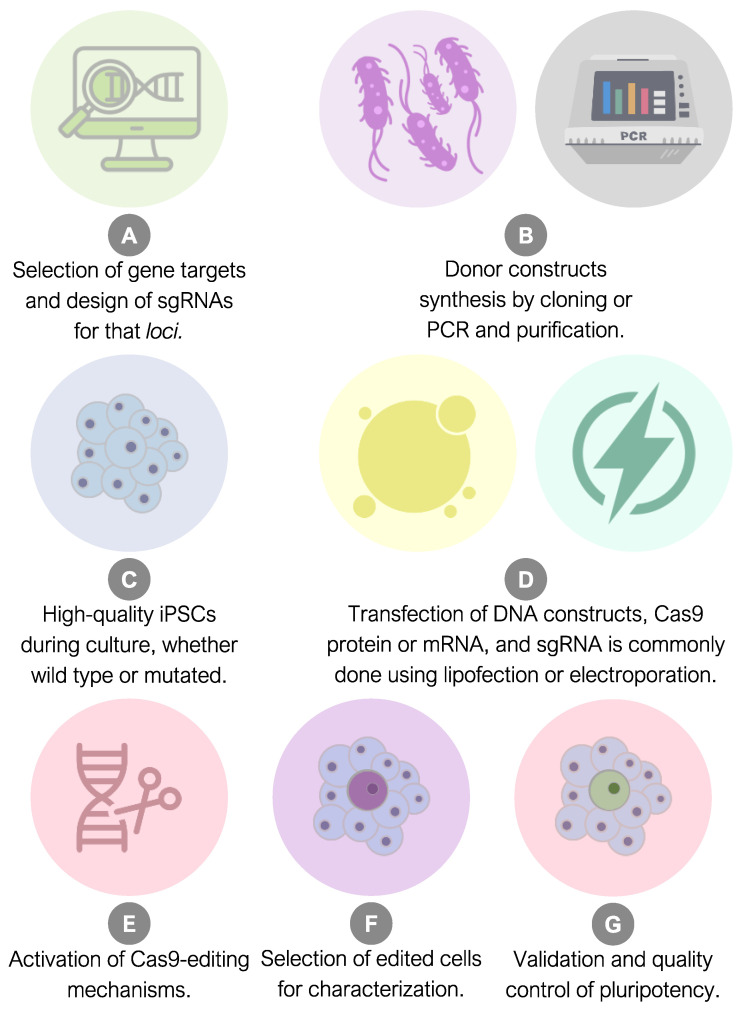
Experimental workflow required for the generation of iPSCs harboring reporter alleles.

To illustrate an experimental workflow for generating iPSCs with reporter alleles using CRISPR-Cas9, we can draw upon the following hypothetical scenario based on the specific literature: First, researchers begin by designing CRISPR-Cas9 constructs targeting specific loci in the genome where the reporter alleles will be integrated (Figure 4A). This design process involves selecting appropriate guide RNAs (gRNAs) that will guide the Cas9 nuclease to the desired genomic sites [115]. The iPSCs are cultured under optimal conditions to maintain their pluripotency (Figure 4C). The CRISPR-Cas9 constructs are then introduced into the iPSCs through delivery systems, such as lipofection, electroporation, or viral transduction [116], which must be standardized for each cell line applied. The CRISPR-Cas9 system is activated within the iPSCs, leading to the generation of double-strand breaks at the target genomic loci (Figure 4D). Concurrently, oligodeoxynucleotides containing the reporter alleles (donor DNA) are provided to facilitate HDR and the insertion of the desired genetic sequences [117]. Following genome editing, the iPSCs are subjected to selection processes to enrich the cells that have successfully integrated the reporter alleles (Figure 4F). This selection step may involve the use of antibiotic resistance genes or other selectable markers to identify and isolate the edited cells [118]. Edited iPSC colonies are then validated to confirm the precise integration of the reporter alleles at the intended genomic sites. Various molecular biology techniques, such as PCR, sequencing, and fluorescence imaging, are employed to verify the presence and expression of the reporter genes [82]. To assess the functionality of the reporter alleles, the edited iPSCs can be differentiated into specific cell lineages, such as neurons or cardiomyocytes, using appropriate induction protocols. The expression of reporter genes can be monitored during the differentiation process to longitudinally evaluate their activity [119] (Figure 4G). Throughout the workflow, quality control measures are implemented to ensure the integrity and stability of the edited iPSC lines. Off-target effects of the CRISPR-Cas9 system can be assessed using cost-effective techniques, like mismatch cleavage assays, or robust sequencing methods, like WGS or GUIDE-seq, to detect unexpected, unwanted, or even adverse alterations to the genome [120]. Finally, the generated iPSC lines with reporter alleles are thoroughly characterized to assess their pluripotency maintenance, genetic stability, and reporter gene expression profiles. Zhong, Li, and Zhou’s 2020 protocol provides comprehensive tips and detailed methods for generating high-quality reporter alleles in iPSCs using CRISPR/Cas9 [121].

### 2.5. Presumable Physiological Impacts of Gene Editing Using iPSCs

In the context of gene transfection using hiPSCs, the goal is to avoid physiological alterations in pathways dependent on the selected gene. Transfection can induce cellular stress, and endonuclease-mediated genome cleavage may lead to apoptosis, making it crucial for researchers to understand these limitations. Strategies like optimizing transfection protocols and using high-fidelity endonucleases can help mitigate these effects. Continuous monitoring of the genetically modified cells is essential for assessing stability and functionality, with routine checks recommended every ten passages, including karyotyping and microarray analysis for chromosomal integrity and gene expression [122,123].

Pluripotency can be evaluated through assays such as teratoma formation, embryoid body induction, and immunofluorescence for pluripotency markers [124,125,126], with the teratoma assay being particularly significant for confirming the presence of all three germ layers [127,128]. However, challenges like off-target effects and maintaining pluripotency after editing persist. CRISPR/Cas9, while powerful, can introduce unintended mutations, raising concerns about genomic integrity [129]. Ongoing research aims to improve the specificity and efficiency of gene editing to enhance the therapeutic potential of iPSCs [130].

## 3. Unveiling Developmental Processes and Disease Mechanisms with Reporter Alleles

### 3.1. hiPSCs and Reporter Alleles in Developmental Biology

The pluripotent nature of iPSCs allows researchers to delve into the mechanisms underlying cell-fate determination and tissue formation, offering valuable insights into developmental principles and lineage commitment [131,132]. By examining the differentiation potential of iPSCs into various cell types, such as neurons, cardiomyocytes, and hepatocytes, scientists can unravel the intricate pathways involved in organogenesis and tissue regeneration [133,134], making it possible to generate complex multicellular structures that mimic aspects of organ development and function [135]. These iPSC-derived structures are called organoids and serve as valuable platforms for studying organogenesis and testing potential therapeutics in a more physiologically relevant context. This whole knowledge about the iPSC developmental dynamics not only enhances our understanding of normal development but also paves the way for regenerative therapies and personalized medicine [136]. In this matter, iPSC technology has advanced the generation of disease-specific models, allowing researchers to replicate developmental defects and investigate the pathophysiology of various disorders [137,138]. By deriving patient-specific iPSCs and differentiating them into relevant cell types, researchers can explore disease mechanisms, screen therapeutics, and develop personalized treatments [119,139]. Additionally, genome editing enables the introduction of specific mutations into normal iPSCs, with the unedited cells serving as isogenic controls. This “developmental mind” guaranteed by the nature of iPSCs has significantly improved our understanding of developmental abnormalities and opened new possibilities for the study of several human conditions [140].

Ashmore-Harris et al. [141] focused on the reporter-gene engineering of hiPSCs during the differentiation process to enable the tracking of hepatocyte-like cells (HLCs) in vivo. The key objective was to develop a method to make the hiPSC-derived HLCs traceable in living organisms. To achieve this, a lentivirus-based gene transfer technique that was reproducible during the differentiation of hiPSCs into HLCs was employed. This approach allowed for the introduction of dual-mode fluorescent and radionuclide reporter genes into the cells, enabling them to be tracked in vitro and in vivo after xenogeneic transplantation. The introduction of the reporter genes did not negatively impact the differentiation process or the maturation of the HLCs. This finding validates the feasibility of using reporter-gene engineering to track cells without compromising their functionality or characteristics. Moreover, the study provided a proof-of-principle for the tracking of hiPSC-derived HLCs in living organisms using single-photon emission computed tomography/computed tomography (SPECT/CT) imaging. The reporter gene imaging technique allowed for the non-invasive monitoring of the labeled cells throughout the body, offering critical information on the behavior and fate of the hiPSC-derived HLCs post-transplantation.

Blöchinger and colleagues [142] published a study on endoderm lineages in 2020. They generated a novel hiPSC line that expresses an *INSULIN-H2B-Cherry* reporter gene. By introducing the H2B-Cherry fluorescent gene under the control of the INSULIN promoter, the researchers developed a tool that provided real-time, non-invasive monitoring of insulin expression during the differentiation of iPSCs into insulin-producing cells. This capability offers a powerful means to investigate the mechanisms underlying human pancreatic β cell development and dysfunction. The INSULIN-H2B-Cherry reporter system not only allows for the visualization of insulin-positive cells but also facilitates their quantification and characterization, which is relevant for various applications in regenerative medicine and diabetes research, opening new possibilities for studying diabetes pathophysiology, drug screening, and cell replacement therapies.

While working on the mesoderm, Gao et al. [143] explored the application of CRISPR/Cas9 technology in creating a triple-fusion reporter gene system for imaging the dynamics and function of transplanted urinary iPSC-derived cardiomyocytes. Their research delved into the use of hiPSCs, derived from renal epithelial cells, as a source for generating cardiomyocytes, aiming to develop cardiac regenerative therapies. The study focused on the development and utilization of a cell line that stably expressed monomeric red fluorescent protein for fluorescence imaging, firefly luciferase for bioluminescence imaging (BLI), and herpes simplex virus thymidine kinase for positron emission tomography (PET) imaging, enabling comprehensive monitoring of the transplanted cardiomyocytes. Beyond that, Galdos et al. [144] reported a *TBX5/MYL2* lineage tracing reporter system to track the differentiation of hiPSCs toward left ventricular cardiomyocytes, particularly from the first heart field (FHF) progenitors. This reporter system allowed for the identification of FHF progenitors and their descendants, providing insights into the predominance of the FHF in the hiPSC differentiation towards cardiomyocytes. By utilizing *TBX5* and *MYL2* to drive the expression of different fluorescent reporters, the researchers were able to trace the lineage of cells originating from the FHF and monitor their differentiation into a specific cardiac cell type, the left ventricular cardiomyocytes. This iPSC lineage tracing system will facilitate significant advances in the study of chamber-specific cardiomyocytes in the context of congenital heart diseases and in the development of novel hiPSC differentiation protocols. In a related 2021 study by Jung et al. [145], a *Brachyury-mCherry* knock-in reporter system was generated in hiPSCs using CRISPR/Cas9 technology. Brachyury, a key transcription factor in mesoderm formation, plays a critical role in the differentiation of mesodermal lineages, including cardiac mesoderm. The introduction of the *Brachyury-mCherry* reporter gene into hiPSCs enabled the researchers to track mesodermal differentiation, providing a real-time tool for visualizing and assessing cell-type enrichment while also shedding light on the dynamics of mesoderm specification and differentiation from hiPSCs.

The following studies focus on the pre-gastrulation process in early embryonic development, specifically the formation of primordial germ cells (PGCs). This process involves cellular commitment within the epiblast [146]. Several research groups are investigating human gametogenesis using iPSCs, often utilizing an intermediate stage known as Incipient Mesoderm-like Cells (iMeLCs) [147]. iMeLCs serve as a transitional phase in the differentiation of hiPSCs toward the germ cell lineage, preceding the formation of human primordial germ cell-like cells (hPGCLCs). hPGCLCs are more specialized cells that closely resemble primordial germ cells. In their 2021 study [148], Kojima et al. used a reporter hiPSC line engineered with *BLIMP1/PRDM1-2A-tdTomato* and *TFAP2C-2A-EGFP* alleles to examine the expression profiles of various cell types, including hiPSCs, iMeLCs, and hPGCLCs, in xenogeneic reconstituted ovaries. This reporter cell line, developed by Sasaki and colleagues [149], enabled the researchers to track and analyze the dynamics of these cells through different developmental stages and experimental conditions. The *BLIMP1* reporter allele facilitated the visualization of cells expressing BLIMP1, a crucial transcription factor in germ cell development. The inclusion of the *TFAP2C-2A-EGFP* allele, which likely marks a downstream target of BLIMP1 signaling, provided a comprehensive view of cellular dynamics and lineage relationships during the differentiation of hiPSCs into germ cell-like cells. This approach offered valuable insights into the molecular mechanisms governing this differentiation process.

The utilization of iPSCs carrying reporter alleles to track neuroectoderm lineages, which include the neural tube and the neural crest lineages, involves specific genes and markers indicative of ectodermal differentiation. Among these markers, NESTIN and PAX6 are frequently used to denote neuroectoderm differentiation [150]. In a significant advancement, Xu and colleagues [151] developed a human embryonic stem cell (hESC) model incorporating a *td-Tomato* reporter at the *PAX6* locus. This innovative approach allows researchers to visually track *PAX6* expression, a crucial step in understanding ectodermal development. Complementing this, Lee et al. [152] generated a *NESTIN-EGFP* reporter hiPSC line, known as KSCBi005-A-1, using the CRISPR/Cas9 technique. NESTIN is a marker strongly associated with neural progenitor cells and indicates early neural differentiation. The creation of the *NESTIN-EGFP* reporter hiPSC line enables real-time monitoring of hiPSCs as they differentiate into neural lineages. The expression of *EGFP*, driven by the *NESTIN* promoter, allows for the visualization of NESTIN-positive cells, facilitating the tracking of neural progenitor cells as they develop into neurons and glial cells. Further enhancing our understanding of neuroectoderm development, Yamashita et al. [153] established a *SOX10* knock-in reporter hiPSC line to visualize neural crest cell differentiation. SOX10 is a critical transcription factor involved in the development of neural crest cells, which differentiate into various cell types in the peripheral nervous system, including neurons, glial cells, and melanocytes. By creating a knock-in reporter system using *SOX10*, researchers can track the expression of SOX10 and observe the differentiation of iPSCs into neural crest cells. This model offers significant insights into the dynamics of neural crest cell specification and migration during embryonic development.

Recent research has made remarkable strides in understanding the surface ectoderm domain, particularly through the work of Inomata et al. [154], who explored the *LGR6* gene function. This gene is expressed in a variety of tissues and is crucial for multiple cellular processes. In the context of skin, LGR6 is linked to hair follicle stem cells [155] and plays a role in bone remodeling and osteogenesis [156,157,158]. Inomata and colleagues [154] specifically investigated LGR6 expression in nail stem-like cells derived from hiPSCs, finding that these cells expressed both *LGR6* mRNA and protein, along with other markers typical of nail stem cells. The *LGR6-GFP* hiPSCs were made to differentiate into limb organoids, and notably, approximately 10 to 30% of these organoids exhibited green fluorescence signals, with the proportion varying depending on the differentiation period. The immunofluorescent staining of the cryosectioned digit organoids revealed co-localization of the GFP and LGR6 proteins. GFP-expressing cells were isolated using cell sorting techniques and subsequently formed spheres, which were injected subcutaneously into the shaved back skin of mice. Twenty days post-transplantation, researchers observed relatively hard lump tissue consisting of both host- and graft-derived cells. These chimeric regions included host-derived vascular endothelial cells and red blood cells. Immunohistochemical analysis further identified cells that were double-positive for human nuclear antigen and GFP, as well as regions that were positive for both KRT17 and KRT81, isotypes of human keratin, highlighting the intricate differentiation capabilities and interactions of LGR6-expressing cells in the development of surface ectodermal tissues.

### 3.2. hiPSCs, Gene Editing, and Reporter Alleles in Disease Modeling

hiPSCs created by reprogramming adult somatic cells into a pluripotent state provide a valuable platform for investigating the molecular mechanisms involved in disease progression [159]. The emergence of gene-editing tools like CRISPR/Cas9 has further amplified the potential of hiPSCs by enabling precise genetic modifications to establish disease models and develop cell-based therapies [160]. Researchers can introduce mutations or correct genetic defects in hiPSCs to investigate their effects on developmental pathways and disease phenotypes [161], offering unprecedented insights into the molecular mechanisms underlying human development and disease phenotypes [162].

The establishment of curated repositories of iPSCs associated with specific diseases, such as Alpha-1 Antitrypsin Deficiency, has been crucial in capturing the phenotypic diversity of patient populations and advancing research in disease pathogenesis [163]. The integration of multi-omics approaches in studying iPSC lines has significantly advanced our understanding of the mutational burden in these cells, enabling better selection of iPSC lines for disease modeling and transplantation therapy [164]. Additionally, gene-editing technologies have enabled the correction of disease-causing mutations in patient-derived iPSCs, offering a platform for generating isogenic control cellular models for studying diseases like osteopetrosis and Huntington’s disease [165,166]. These advancements have been pivotal in the development of targeted therapies.

On top of this all, reporter allele technology, an application of gene-editing platforms previously discussed, enhances the utility of hiPSCs [167]. With reporter alleles, researchers can monitor the activation of developmental pathways and pinpoint crucial stages in cell lineage specification and differentiation. In disease models, these reporters offer insights into the impact of mutations or pathogenic conditions on cellular functions and the dynamics of gene expression. This method aids in identifying potential therapeutic targets and evaluating drug efficacy under controlled, reproducible conditions. Ultimately, the integration of reporter alleles in hiPSCs facilitates a seamless connection between fundamental research and clinical applications, enriching our comprehension of intricate biological processes and disease mechanisms. To illustrate this, we trace the evolution from the early days of gene targeting in hiPSCs to recent studies.

In 2009, Hockemeyer and colleagues published one of the pioneering studies on reporter alleles in human ESCs and iPSCs [168]. Their research focused on the ZFN-mediated gene targeting of two distinct genes in these cell types. One commonly used reporter in their study is GFP expression driven by the *OCT4* promoter, which marks undifferentiated pluripotent stem cells. This system has been utilized to monitor the maintenance of pluripotency during the culture and differentiation of human ESCs. Additionally, they generated reporter cell lines of human ESCs and iPSCs for *PITX3*, a gene activated during differentiation into dopaminergic neurons. This approach demonstrated that non-expressed genes could also be targeted, which is beneficial for leveraging the differentiation capabilities of pluripotent stem cells. Zou et al. [169] conducted a trailblazing study using a virus-free system to achieve ZFN-enhanced gene targeting of the endogenous *PIG-A* gene with *GFP* reporter gene in both human ESCs and iPSCs. The *PIG-A* gene is crucial for the retention of multiple glycosyl-phosphatidyl-inositol-anchored proteins (GPI-APs) on the cell surface and is mutated in hematopoietic stem cells from patients with the blood disorder paroxysmal nocturnal hemoglobinuria (PNH). The study successfully demonstrated the gene targeting of an endogenous gene in human PSCs, underscoring the potential of this technique in disease-specific hiPSCs for both research and clinical applications.

Over the years, while maintaining a focus on disease modeling, therapeutic interventions, and the potential applications of cell therapies, Calatayud et al. [95] utilized CRISPR/Cas9 technology to develop the tyrosine hydroxylase (TH) reporter hiPSC line. These cells express *mOrange* as a fluorescent marker inserted at the last exon of the *TH* gene, enabling live imaging and isolation of dopaminergic neurons crucial for studying Parkinson disease (PD) research and other neurodegenerative disorders. By deriving iPSCs from patient-specific cells and guiding them to become TH-positive dopaminergic neurons, the study replicated disease-relevant cell types in vitro, facilitating investigations into PD pathogenesis and screening potential therapies. Unlike previous methods that often yielded mixed neuronal cultures [170], this CRISPR/Cas9 approach produces a homogeneous population of TH-positive dopaminergic neurons. The *mOrange* reporter faithfully mirrors native *TH* expression, facilitating direct visualization and isolation of TH-expressing cells in mixed cultures. Additionally, the study employed calcium imaging to distinguish functional differences between the dopaminergic and non-dopaminergic neurons in the ventral midbrain, enhancing both live-imaging capabilities and the ability to purify specific cell populations using fluorescence-activated cell sorting for further molecular and functional analyses.

Another important goal is to generate early cardiomyocytes of specific regions of the heart. The study by Bizy et al. [171] explores the use of a human ventricular myosin light chain (*MYL2*) reporter construct created using homologous recombination to identify early ventricular cardiac myocytes derived from hiPSCs. They found that MYL2 expression is a reliable marker for hiPSCs committed to the ventricular lineage, enabling the hiPSC-derived cardiac myocytes differentiation with an early ventricular phenotype. This finding is crucial for regenerative medicine as it offers a method to specifically isolate and identify early ventricular cardiac myocytes from hiPSCs. In the same vein, Chirikian and colleagues, in 2021, utilized the CRISPR/Cas9 system to introduce fluorescent reporters into hiPSCs, enabling the isolation of atrial- and ventricular-specific cardiomyocytes (CMs) [172]. This research addresses a key challenge in current differentiation protocols, which often yield heterogeneous cell populations predominantly composed of ventricular-like CMs. To overcome this, two chamber-specific reporter hiPSC lines were developed. In the *MYL2-tdTomato* reporter line, the red fluorescent *tdTomato* was inserted just upstream of the 3′ untranslated region of the *MYL2* gene, effectively labeling ventricular-like CMs without interfering with the endogenous gene function. Similarly, the *SLN-CFP* reporter line integrated Cyan Fluorescent Protein (CFP) downstream of the coding region of the atrial-specific gene Sarcolipin (*SLN*). These modifications allowed the researchers to simultaneously purify tdTomato+ and CFP+ CMs derived from a single hiPSC line using flow cytometry. This advancement is significant for cardiac research, as it provides a precise method for isolating atrial- and ventricular-specific CMs from hiPSCs, which is essential for studying heart development and disease modeling, facilitating the precise isolation and characterization of specific cardiac cell types for therapeutic applications.

Table 1 provides a concise expansion of the topics covered in the preceding paragraphs, presenting key studies in disease modeling and understanding the foundations of critical developmental processes relevant to disease contexts.

Another significant application of the reporter alleles in hiPSCs merges concepts and insights from developmental biology and disease pathogenesis. This application focuses on the advanced study of drugs and the identification of novel therapeutics for patients. As we previously discussed, the utility of hiPSCs in drug screening is especially notable because they favor the formation of multicellular spheroids or organoids and can produce unlimited disease-specific cells from patient-derived samples, transforming traditional drug-development methods by enabling a personalized approach to assessing drug efficacy and toxicity [190]. hiPSCs have shown to be superior to hESCs and animal models in disease modeling, drug screening, and cell replacement therapy, highlighting their effectiveness in advancing pharmacological discovery [191]. For instance, the use of patient-specific hiPSCs for ex vivo modeling of cardiovascular disorders has proven their potential in cardiovascular regenerative medicine, demonstrating the versatility and effectiveness of hiPSC technology in therapeutic advancements [192]. This becomes evident when we consider the numerous studies focused on cardiovascular research, as discussed in previous paragraphs. However, significant gaps remain, and the number of studies utilizing reporter alleles for drug development, toxicity testing, and other areas is still limited. Most reports on iPSC-based compound screens utilize targeted screening approaches with a limited number of known or predicted disease modulators. This likely reflects the inherent challenges in generating disease-relevant cell types at high scale and purity for larger-scale screening efforts. Therefore, more research is necessary to fully harness the potential of hiPSC reporter lines and 3D cell culture in advancing pharmacological discovery and ensuring their effective application in these fields.

## 4. Conclusions

The integration of reporter alleles into hiPSCs using several gene-editing technologies has revolutionized developmental biology, disease modeling, and pharmaceutical research. Precisely incorporating reporter genes into specific hiPSC loci enables continuous monitoring of cellular differentiation and fate, both in vitro and in vivo post transplantation. These reporter hiPSCs maintain essential pluripotent stem cell properties, facilitating detailed investigations into the developmental processes and the effects of genetic mutations on cellular behavior. Additionally, introducing reporter alleles into hiPSCs from patients with specific disease phenotypes allows dynamic tracking of genetic corrections using CRISPR/Cas9 or other platforms, ensuring precise mutation correction and simplifying the creation of isogenic controls for disease modeling. Notably, lineage-specific reporters in hiPSCs can play a key role in enriching or isolating differentiated cells, which is particularly important in preclinical in vivo studies. This ensures that no uncommitted cells remain in the cell pool, which could otherwise lead to tumor development after injection or transplantation. Standardized reporter hiPSC lines, rapidly generated and validated through several protocols discussed here, form a robust foundation for creating databases that catalog drug responses and mutation effects across various cell types with consistent genetic backgrounds. This advancement significantly enhances iPSC-based drug screening and discovery efforts.

In the future, ameliorated gene-editing techniques focused on creating reporter cell lines within hiPSCs are necessary for substantial advancements. Emerging technologies, like base editing, prime editing, and CRISPR-based multiplexing, promise to fill this gap and streamline the creation of sophisticated reporter systems. These systems not only monitor gene expression dynamics but also track cellular differentiation and response to external stimuli with unprecedented accuracy. Innovations in synthetic biology, including AI and machine learning, as well as the extrapolation of CRISPR systems and advanced delivery methods, are poised to expand the repertoire of available reporter cell lines, facilitating their application across diverse research and clinical settings. As these methodologies evolve, the future of reporter cell lines in hiPSCs appears increasingly promising, steadily driving breakthroughs in both fundamental biology and therapeutic development.

## Figures and Tables

**Table 1:** Key studies utilizing reporter alleles in hiPSCs for modeling development and phenotypes of various diseases.

Purpose	Reporter Allele	Platform Used	Reference
Temporal and partial inhibition of glioma-associated oncogene 1 exerts oligodendrocyte induction.	*OLIG2-GFP*	Homologous recombination	[173,174]
Detecting the reactivation of silenced *FMR1* in cells of patients with fragile X syndrome.	*FMR1-Nluc*	CRISPR/Cas9	[175]
Track the specification of trophectoderm in early embryogenesis.	*CDX2-Venus*	TALENs	[176]
Modeling amyotrophic lateral sclerosis and frontotemporal dementia by tracking stress granule dynamics in *P525L* mutated cells.	*FUS-GFP*	CRISPR/Cas9	[177]
Function of β pancreatic cells and developing motor neurons.	*NKX6.1-GFP*	CRISPR/Cas9	[178]
Purification of smooth muscle cells for the study of muscle physiology and mechanics.	*ACTA2-GFP*	CRISPR/Cas9	[179]
Cartilage tissue engineering for regenerative therapies for joint injuries and diseases.	*COL2A1-GFP*	CRISPR/Cas9	[180,181]
Function of α pancreatic cells.	*ARX-CFP*	CRISPR/Cas9	[182]
Development of myogenic precursors of satellite cells.	*PAX-7-Venus*	CRISPR/Cas9	[183]
Modelling human chondrodysplasias caused by mutations in *TRPV4.*	*SOX9-tdTomato*	CRISPR/Cas9	[96]
Study of liver fibrosis progression using hepatic stellate cells derived from hiPSCs.	*ACTA2-RFP*	CRISPR/Cas9	[184]
Track the cardiac differentiation process.	*NKX2-5-GFP*	TALENs	[185]
Modelling myriad disorders in patients with Down syndrome.	*AAVS1-GFP*	ZFNs	[186]
Purification of serotonin-productive neurons for the study of neuropsychiatric diseases.	*TPH2-GFP*	CRISPR/Cas9	[187]
Study and therapeutic application for skeletal muscle function.	*ACTA1-tdTomato*	CRISPR/Cas9	[188]
Target insulin influence on the differentiation process of endocrine pancreatic cells.	*INS-Cherry*	CRISPR/Cas9	[189]
